# Editorial to Special Issue “Sleep Apnea and Intermittent Hypoxia 2.0”

**DOI:** 10.3390/ijms23105299

**Published:** 2022-05-10

**Authors:** Shin Takasawa

**Affiliations:** Department of Biochemistry, Nara Medical University, 840 Shijo-cho, Kashihara 634-8521, Japan; shintksw@naramed-u.ac.jp; Tel.: +81-744-22-3051 (ext. 2227); Fax: +81-744-24-9525

Sleep apnea syndrome (SAS) is the most common form of sleep-disordered breathing and is associated with many adverse health consequences, including increased overall mortality risk. It is estimated that nearly 1 billion adults worldwide aged 30–69 may suffer from SAS [1]. SAS is characterized by a repetitive partial or complete upper airway collapse during sleep, resulting in altered alveolar ventilation, intermittent hypoxia (IH) along with increased respiratory efforts, and intrathoracic negative pressure swings that frequently lead to arousal, therefore disturbing sleep continuity and resulting in fragmented sleep architecture. It induces apnea and hypopnea, which often result in decreased oxygen saturation. A growing body of evidence suggests that SAS acts through recurrent episodes of oxygen desaturation and reoxygenation, which cause diabetes, hypertension, stroke, ocular complications, cognitive impairment, and ischemic heart disease [2].

In 2019, the Special Issue of the *International Journal of Molecular Sciences* (*IJMS*), “Sleep Apnea and Intermittent Hypoxia”, covered several aspects of SAS and IH [3,4,5,6,7,8,9,10]. To continue the previous Special Issue, the second volume, “Sleep Apnea and Intermittent Hypoxia 2.0”, explores more insights into SAS and IH and collected seven publications that consist of two original research articles and five literature reviews. All seven articles have brought a new wind in the research of SAS and IH from individual viewpoints and will encourage attractive approaches by future researchers.

Hypertension is the most common complication of SAS. SAS, as a risk factor for hypertension, has been extensively studied previously [11]. The pathophysiology of the interaction is based on the dysregulated activity of the sympathetic nervous system and renin-angiotensin-aldosterone system [11]. In the Special Issue, the upregulation of renin together with Cd38 (ADP-ribosyl cyclase/cyclic ADP-ribose [cADPR] hydrolase) [12] upregulation in response to IH in juxtaglomerular cells was reported [13]. The upregulation of renin was attenuated by the addition of a cell-permeable cADPR antagonist, 8-bromo-cADPR, in cell culture medium, suggesting that renin expression is regulated via the CD38-cADPR signal system. The upregulation of renin and Cd38 was caused by the downregulation of microRNA-203 but not by transcriptional activation of the gene(s). In SAS therapy, continuous positive airway pressure (CPAP) is the most common treatment, and it works by generating airway patency, which will counteract the apnea or hypopnea. Baran et al. [14] compared several clinical trials of SAS and hypertension and concluded that the best treatment of SAS patients with hypertension is a combination treatment of antihypertensive medication and CPAP therapy, which have respectively demonstrated a significant additive effect on blood pressure and an improved well-being of SAS patients. SAS is also known to be an independent cardiovascular risk factor, and numerous studies on the pathophysiological mechanisms of SAS contributing to an increased cardiovascular risk have been conducted. Mochol et al. [15] reviewed the effects of CPAP on changes in the profile of the endothelial and blood component functions and its subsequent potential clinical advantage in lowering cardiovascular risk in other comorbidities, such as diabetes, atherosclerosis, hypertension, atrial fibrillation, and stroke. Current evidence demonstrates the activation of an inflammatory pathway in circulating monocytes by IH, the important characteristic of SAS, which is a critical step that induces injury to the endothelium. The adhesion and transmigration of monocytes through the vascular endothelial layer are initiated by the attraction by chemokines, resulting in the development of atherosclerosis. Chuang et al. [16] demonstrated that IH can enhance the interleukin (IL)-8 gene expression, protein secretion, and the subsequent chemotactic migration ability of monocytes and that monocytic IL-8 expression in SAS patients was elevated after one night’s sleep and was positively dependent on disease severity. The findings pointed out the important role of IL-8, which is responsible for the increased chemotactic migration of monocytes under IH condition, suggesting that blocking the IL-8 function with antagonists to reduce the IH-induced chemotactic migration of monocytes, an early inflammatory process of atherosclerosis, could be one potential strategy to reduce the progression of atherosclerosis in patients with SAS.

Next to hypertension, diabetes, insulin resistance, and obesity are important complications in patients with SAS. Uchiyama et al. [17] pointed out and discussed cytokine expression in hepatocytes, adipocytes, and skeletal muscle myocytes, in which hepatokines, adipokines, and myokines are expressed. In particular, selenoprotein P expressed in hepatocytes; tumor necrosis factor-α, C-C motif chemokine ligand 2, and resistin expressed in adipocytes [6]; and IL-8, osteonectin, and myonectin expressed in skeletal muscle myocytes, which are known as diabetes/insulin-resistance-prone cytokines, were revealed to express in response to IH stimulation. Obesity is a major risk factor for SAS, as well as metabolic syndromes such as diabetes and insulin resistance. In fact, obesity is one of the most important risk factors for the development of SAS, and more than 70% SAS patients are obese. On the other hand, it was reported that 20% of adult SAS patients were not obese. Weight gain generally results from excessive food intake driven by an excessive appetite, leading to a positive energy balance. Appetite is regulated by both the hypothalamus and the gut, as a gut–brain axis driven by differential neural and hormonal signals. Shobatake et al. [18] pointed out and discussed the anorexigenic effects of IH on the gut–brain axis by the IH-induced upregulation of proopiomelanocortin and cocaine- and amphetamine-regulated transcript in neuronal cells as well as the IH-induced upregulation of peptide YY, glucagon-like peptide-1, and neurotensin in enteroendocrine cells [5]. Changes in both the neuronal and enteroendocrine cells in IH suggest possible anorexigenic effects of IH on the gut–brain axis.

SAS patients present with a higher prevalence of age-related disorders, such as hypertension or diabetes, and SAS patients are associated with telomere shorting. Turkiewicz et al. [19] summarized study outcomes on changes in leukocyte telomere length in SAS patients and described possible molecular mechanisms that connect cellular senescence and the pathophysiology of SAS.

As shown in Figure 1, there exist differences between human SAS patients and in vitro and in vivo experimental models. In addition, a number of population-based studies have shown that SAS is more common in men than in women (male:female ratios ranging from 3:1 to 5:1 in the general population), and there are a number of pathophysiological differences such as differences in obesity, upper airway anatomy, breathing control, hormones, and aging to suggest why men are more prone to SAS than women [20,21]. However, these differences have not been fully elucidated, and therefore why SAS is more common in men than in women is still elusive. The problems will hopefully be solved by future enriched research from both basic and clinical viewpoints. Therefore, the third volume, “Sleep Apnea and Intermittent Hypoxia 3.0”, will start to explore more insights into the SAS and IH.

Finally, the Editor is delighted to have had the honor of organizing this Special Issue for *IJMS*, which highlights the research of eminent scientists in the field of SAS and IH. The Editor would like to thank all the contributors to this Special Issue for their commitment and enthusiasm during the compilation of the respective articles. The Editor also wishes to thank Tinsley Qiu and other members of the editorial staff at Multidisciplinary Digital Publishing Institute (MDPI) for their professionalism and dedication. Hopefully, readers will enjoy this Special Issue and be inspired with new ideas for future research. Based on the two Special Issues, “Sleep Apnea and Intermittent Hypoxia 3.0” is in progress. The Editor sincerely looks forward to the submission of original papers and reviews on SAS and IH.

## Figures and Tables

**Figure 1:**
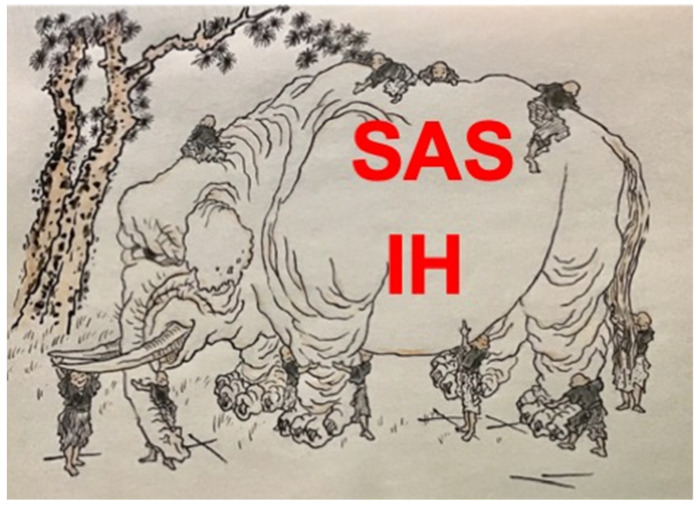
The Blind Men and the Elephant (by Ktsushika Hokusai [1760–1849]) is modified to depict our understanding of SAS and IH. “And so these men of Indostan. Disputed loud and long, Each in his own opinion. Exceeding stiff and strong, Though each was partly in the right. And all were in the wrong!” (“The Blind Men and Elephant” by John Godfrey Saxe [1816–1887]). It may be difficult for us to immediately grasp the essence and the whole picture of SAS and IH, but let us do our best to believe that we will reach comprehension of the essence and the whole picture by continuing efforts to accumulate new understanding step by step. Is it not?
